# Supplementary material for: Low-Cost Devices for Three-Dimensional Cell Aggregation, Real-Time Monitoring Microscopy, Microfluidic Immunostaining, and Deconvolution Analysis
Source: Bioengineering (Basel). 2022 Feb 3;9(2):60. doi: 10.3390/bioengineering9020060 (PMC8869754; doi:10.3390/bioengineering9020060)
Supplement: Supplementary file 1 [file bioengineering-09-00060-s001.zip › bioengineering-1547087-supplementary.pdf]

bioengineering

Supplementary Materials:

Supplementary 1: Teflon Hanging Drop Devices (THDD)

The design of the hanging drop chip was created with the freeware 2D-CAD program LibreCAD (librecad.org). The file was saved as dxf-file and then opened with the freeware Estlcam (www.estlcam.de). This software allows setting various parameters such as the position of drilling location and the milling depth, which in the here described case was set to 3.2 mm. The file has to be saved as an nc-file. The 2-mm thick poly-*tetra*-fluoroethylene (Teflon) plate was processed with the aid of the Qbot MiniMill (www.minimill.at) and the nc-file was opened with the MiniMill program. After completion of milling, the plate was trimmed to the appropriate size with the help of a Trotec speedy-100 CO_2-laser cutter.

To warrant well-aimed drop transfer from the hanging drop device into the TµWs, a gage device was constructed (Figure S1). The chip holder for the hanging drop chip was designed with FreeCAD (www.freecadweb.org). The chip holder was printed with 3D printer: Prusa i3 MK3 (Prusa, Czech Republic) using Vertex, 3D Printing Filament, PLA175Z07, purple, diameter 1.75 (Velleman, Belgium). The holder supported injecting cell suspension through the holes of the Teflon chip; it also supported lossless transfer of the drops from the hanging drop chip into the staining chip.

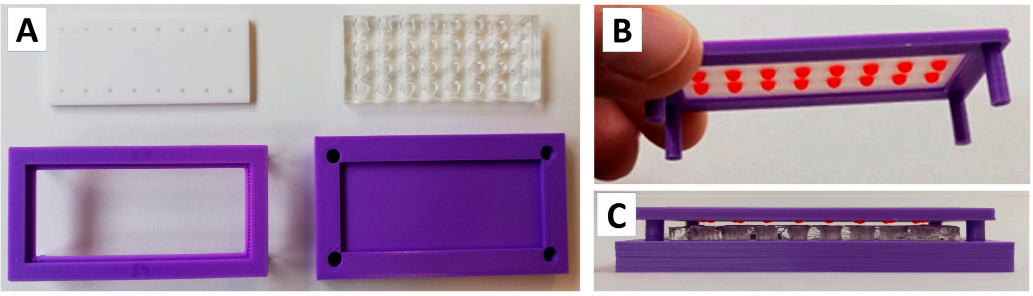

Figure S1. Chip Holder for Teflon Hanging Drop Devices (THDD): Chip holder for positioning a hanging drop chip above a staining chip (**A**) enabling overflow transfer (**B**) and exact falling off of drops into the corresponding chambers (**C**).

A humidified incubation chamber (HIC) with glass slides at the top and the bottom of the chamber for microscopic observation of spheroid building and a water reservoir for stable humidity inside the chamber was designed with AutoCAD (Autodesk, USA) and additively manufactured with the help of the 3D-printer: Prusa i3 MK3 using Renkforce Metal Filled Filament, Color: Copper, 20% Bronze powder, No. 152 83 44 (Conrad Electronic SE, Austria). The following CAD files were needed to build the devices for the THDD:

- Frame_Hanging_Drop_Chip.dxf
- Milling_Hanging_Drop_Chip.nc
- Holder_Hanging_Drop_Chip.stl
- Incubation_Chamber_Bottom_Part.stl
- Incubation_Chamber_Chip_Holder.stl
- Incubation_Chamber_Lid.stl

Supplementary 2: Twin-microwell Staining (TµW)

Two types of staining chips were built, TµW-PDMS (Figure S2A) and TµW-PMMA (Figure S2B).

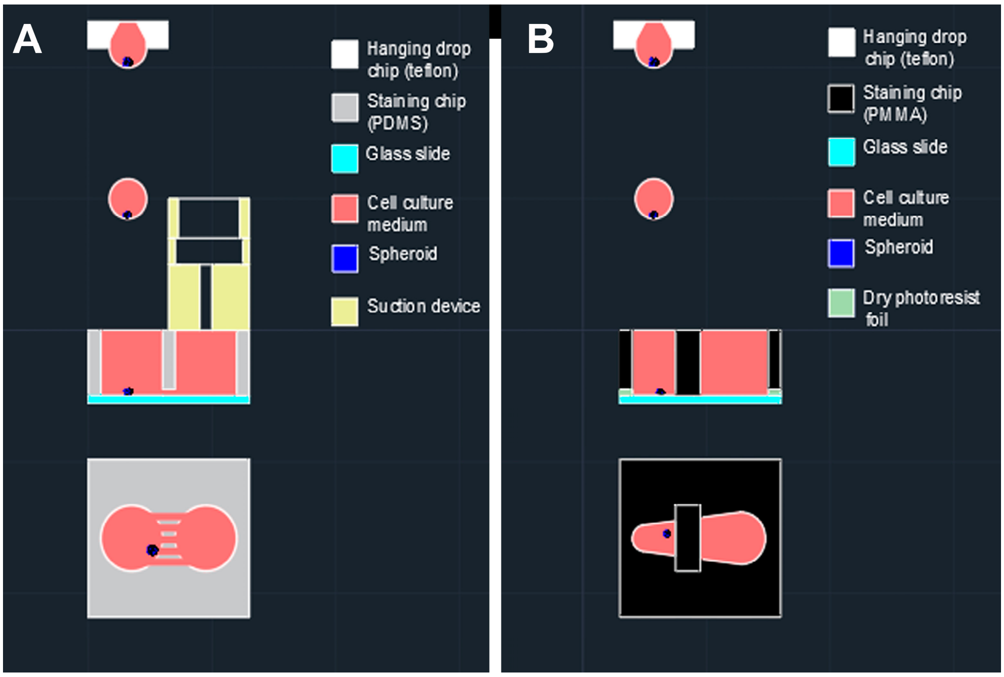

Figure S2. Twin-microwell Staining (TµW) Devices: (**A**): Schematic illustration of staining chips made of PDMS; (**B**): schematic illustration of staining chips made of PMMA. Chip design, function and workflow are depicted. Upper left side shows a cell aggregate-forming drop hanging from a Teflon chip; next the drop falls off and settles into a twin-microwell through its inlet (central part of both figures); as depicted in the lower part of the panels, suspending fluids can be exchanged through connecting channels (PDMS version), or the slot system (PMMA version).

The layout for both devices were designed with LibreCAD. TµW-PDMS was made of PDMS. A photomask was manufactured with the help of the Filmstar photoplotter (Bungard, Germany). The black and white photomask (Agfa, Belgium) was applied to generate 3D SU-8 microstructures as a mold for subsequent PDMS casting. A 100-mm silicon wafer (Siegert Wafer, Germany) was coated with a 60 µm high SU-8 2035 layer (Microchem/Kayaku, Japan). The photomask was used for UV curing of the SU-8 2035 layer. Therefore, the photomask was put on the uncured SU-8 2035 layer and then it was UV cured for 7 s in the UV cube (UV-Kub 2, France). The uncured SU-8 was removed with SU-8 developer (Microchem/Kayaku, Japan). Then the reaction was stopped with isopropanol (Merck, Germany). PDMS was mixed by using 9 parts of Nusil MED-6015 Part A and 1 part Nusil MED-6015 Part B (Avantor, PA USA). In order to remove bubbles, which emerged during mixing, the PDMS was placed in a desiccator for degassing. A 5-mm high PMMA frame was glued on the SU-8 wafer to form a mold for PDMS casting inside the frame. To accelerate curing of PDMS, the SU-8 wafer was placed on a hot plate at 95 °C. After approx. 45 min the PDMS hardened, thus the elastomer could be cut out of the frame with a scalpel. Wells were punched out of the PDMS slab with a 5-mm biopsy-punch tool using a template made of PMMA to warrant equal distances between the holes. The PDMS was then plasma-bonded on a glass slide, which serves as the bottom of the chip. These wells are connected through small channels with a channel height of either 60 µm and a channel width of 100 µm or a channel height of 60 µm and a channel width of 400 µm. The channels shall form a barrier for the cell aggregates thus preventing slipping through during exchange of solutions. The wells on the outer edges of the chip accommodated the cell aggregates while the well located inside were used to deliver buffers and reagents. To

ease removal of solutions, a suction device was designed with LibreCAD and built out of PMMA, which can be connected to a water jet pump or to an evacuated pump collection barrel.

The following files were needed to build the twin microwell staining device of PDMS:

- Staining_Chip_PDMS.dxf
- Template_Biopsy_Punch.dxf
- Holder_Staining_Chip.stl

TμW-PMMA consisted of 3-mm thick PMMA. This chip contained chambers, which were separated by a slider forming a 50-μm vertical gateway at the bottom allowing efficient solution exchange, while the specimen stay in place. The PMMA chip and the 16-sliders were laser-cut. A portable 30 μm thick PCB Photosensitive Dry Film (Mungolux, Germany) for Circuit Production was used to bond the PMMA to a glass coverslip. Before cutting the PMMA chip the dry film photoresist was taped onto the PMMA and then the chip was cut. PMMA chip and glass slide were pressed together and dried on a heating plate for 1 min at 110 °C.

The following file was needed to build the mounting device of PMMA:

- Staining_Chip_PMMA.dxf

Supplementary 3: Auxiliary Devices for in chip fluid exchange

Suction device for TμW-PDMS: In order to ease handling when performing spheroid staining in TμW-PDMS, a suction device was designed. When connected to a water jet pump, it can be used for discarding medium instead of using a pipette. This suction device consists of 3 PMMA parts, which were glued together with double-sided adhesive tape (Figure S3).

The following file was needed to build the suction device:

- Suction_device.dxf

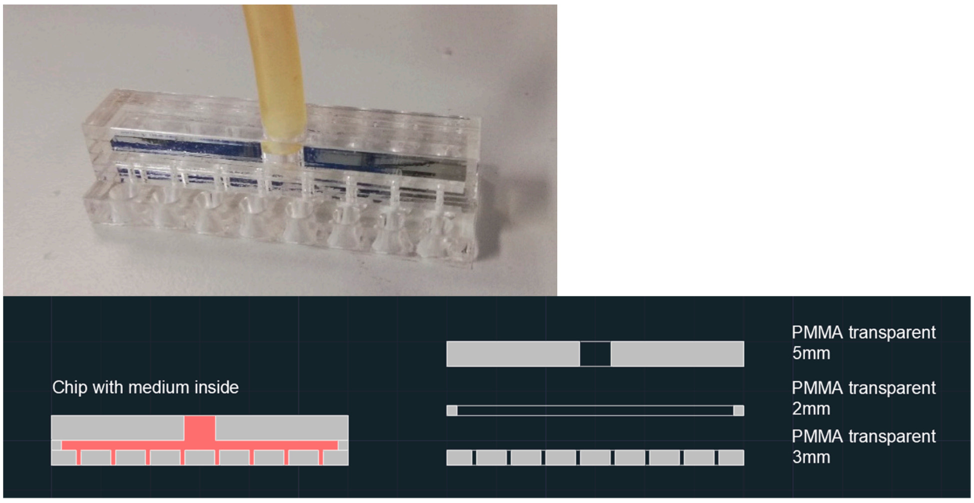

Figure S3: Auxiliary Devices for in chip fluid exchange: Illustration of the suction device, which consists of 3 parts glued together with double-sided adhesive tape. The lower part consists of 16 vertical channels that when placed exactly over the wells of TμW-PDMS aspirate the liquid from the twin-microwells. The middle part consists of a chamber and the upper part has a round cut-out in the middle to which the tube of a water jet pump can be connected.

Supplementary 4: Delta-kinematic microscope stage

Based on a delta kinematic design, the created device has a small footprint, which eases placement in incubators. The kinematic was controlled via python, thus making it amenable for automated observing, manipulating and monitoring of appliances and events at the microscale over prolonged time periods.

Materials and Technology

Computer-aided design (CAD) in combination with computer-aided manufacturing (CAM) technology was applied to generate and 3D print the load-bearing parts. The design was achieved with the aid of the open-source CAD software, openSCAD (openscad.org).

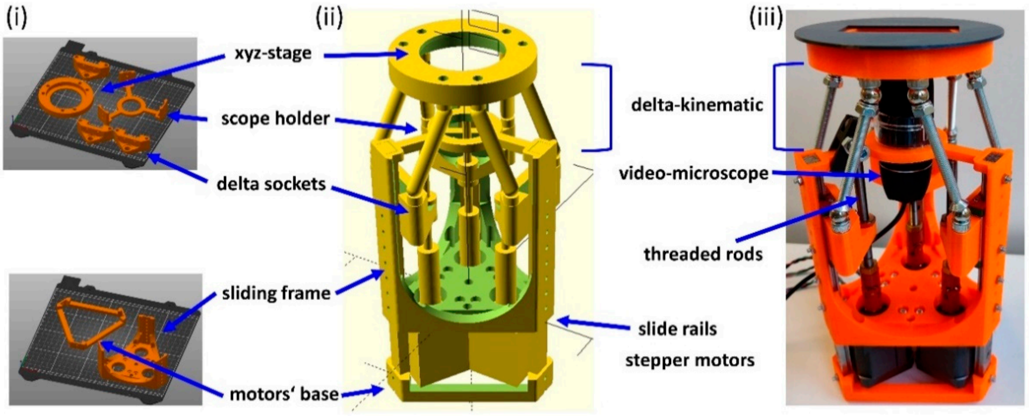

Figure S4. Delta kinematic microscope stage: Delta kinematic stage, (i) individual parts as depicted after slicing before 3D printing, (ii) in a fully integrated CAD view and, (iii) completed holding a video microscope (right)

For the individual building parts, files in stl format were exported and translated into a format, which could be interpreted by 3D printers (Prusa Slicer, Pursa3d.com), in our case the 3D filament printer Prusa MK3. The respective files (stl as well as the sliced format are freely available for download at https://github.com/spoc-lab/delta-microscope/tree/master/files/3d The STL files for the robotic stage were sliced in Prusa Slicer 2.2.0 Figure S4 and printed in ecoPLA NeonOrange with 20% infill.

Materials used for assembly were as compiled in the table S1 and are depicted in Figure S4.

Table S1. Building Parts for Delta-kinematic Stage.

Item	Number	Purpose	Company *	Part-No
Allegro A4983 Based Stepper Boards: here the ‘Big Easy Driver’	3	stepper board	Technobots	2900-445
Teensy 4.0 or 4.1 Microcontroller	1	stepper control	Conrad	2269230
Nema 17 Steppers (0.9° 2.4 A)	3	stepper motor	Act-motor	1402-050
Fine Hex Adjuster, 1/4”-80, 4” Long	3	leadscrew	Thorlabs	F25SS400
Locking Phosphor-Bronze Bushing with Nut, 1/4”-80, L=0.50”	3	leadscrew nut	Thorlabs	N80L6P
Linear Guide Rails (min 200 mm)	3	slides platform	Amazon	CNBTR214
Universal Coupling Body	3	motor-screw-connector	Technobots	4604-050
Universal Coupling Insert – 5 mm	3	motor-side	Technobots	4604-059
Universal Coupling Insert - 1/4”	3	screw-side	Technobots	4604-066
OTHER SMALL ITEMS				
160 mm x 100 mm Copper Clad Stripboard	1	mount for electronics	Conrad	
Male & Female PCB Headers 2.45-mm pitch	~10	simple connectors	Conrad	
Screws M3 (10 mm): 12 for motors, 6 for sliders, 15 for guides	33	mount slider & motors	Bauhaus	
Nuts M3	33	fixing screws	Bauhaus	
Screws M6 (15-mm)	12	suspension	Bauhaus	
Screws M6 (60-mm)	6	suspension	Bauhaus	
Nuts M6 (end nut)	6	suspension	Bauhaus	
neodymium bullet magnets ⌀ 10 mm	6	suspension	Bauhaus	
USB (micro) cable	1		Conrad	
6 wire colored ribbon cable	3 m		Conrad	
12 V / >=5 A power supply	1		Conrad	
197 g 3D-printer filament (PLA 1.75-mm)	~250 g	microscope structure	3d jake	
USB Digital Microscope 40x-1000x	1	microscope camera	Bysameyee	

* Technobots: www.technobotsonline.com; Thorlabs: www.thorlabs.com; Conrad: www.conrad.at; Bauhaus: www.bauhaus.at; 3djake: www.3djake.at; Bysameyee: www.bysameyee.com/microscope

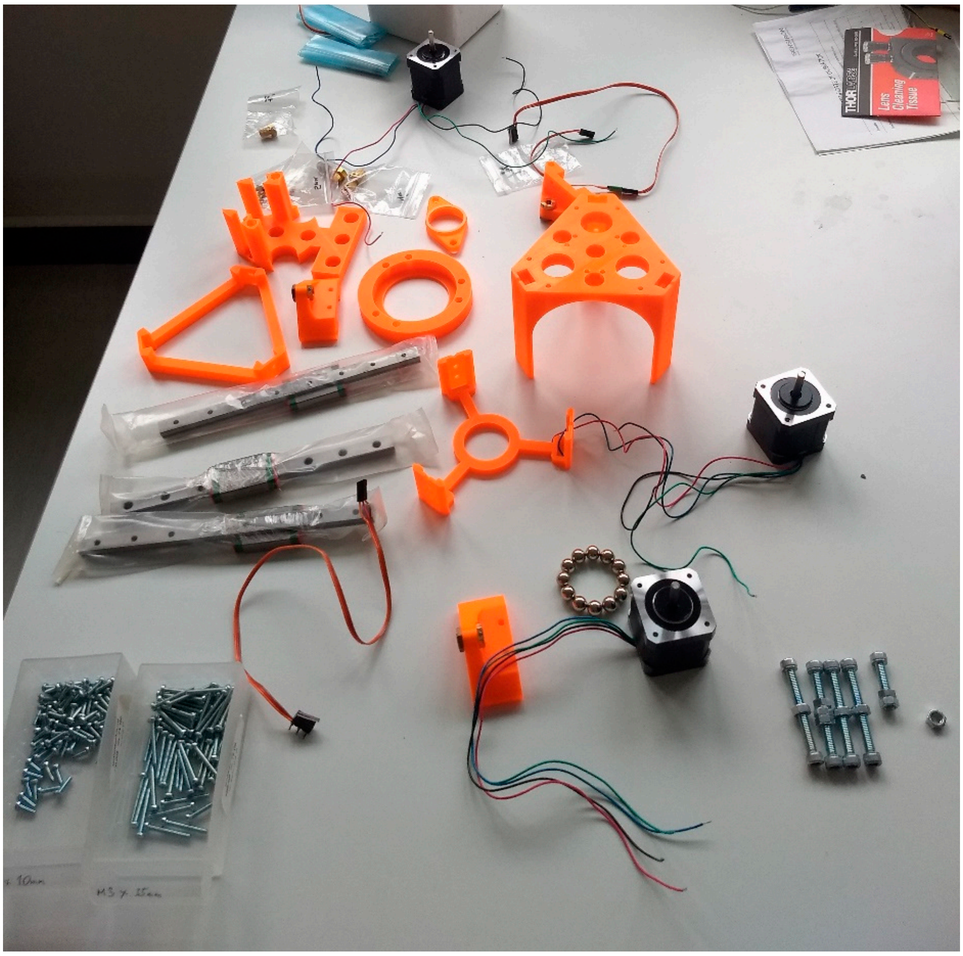

Figure S5. Building Parts for Delta-kinematic Stage: Parts before assembly

Mechanical Assembly was accomplished by first tinning the leads of the motors with solder, then adding a male PCB-connector (or any other connector), next using heat-shrinking tube to electrically isolate the connectors at the end, and eventually adding the mechanical connector for the leadscrew (5-mm brass insert) to the motor as one is greatly hindered later on in assembly space-wise. For the leadscrew part, the brass inserts are added to connect the motor onto the leadscrew, the screws are inserted very carefully to avoid damaging the thread.

Assembly

The printed objects Figure S5.and the parts in Table S1 were assembled as follows:

(1) The linear guide rails were screwed onto the main part, and the top part was then screwed onto the guide rails with 15xM3 screws and nuts. For correct assembly, the top part has to end on height of the guide rails.

(2) The three stepper motors were then screwed (12xM3) onto the bottom part. The bottom part needed to be screwed on the other end of the guide rails (3xM3). Now the universal couplings were screwed onto the motors and the optical fine threaded rods were fixed on the other side of the universal couplings.

(3) For better durability, the plastic part of each universal coupling was glued to the metallic part with epoxy glue. Six M6 screws were glued into the three slider parts, and 6xM6 were glued into the platform.

(4) The slider parts were joined with their threaded insert and screwed onto the guide rails.

(5) The end nuts were placed on the 60-mm M6 screws and the microscope stage was completed by putting the neodymium magnets in the joint positions.

(6) Finally, the microscope-camera was put in the ring on top where it is held in position with the printed clamp.

Controls

An Allegro 4983 stepper driver chip was implemented (Allegro Microsystems), in order to control the stepper motors. We used the 'Big Easy Driver' for each motor (https://www.sparkfun.com/products/12859) as the latter works by default in a 16-step microstepping mode ('MS1, MS2, MS3' pins unconnected). The stepper drivers were controlled with a teensy 4.0 or 4.1 microcontroller.

Assembly of the board was as follows:

(1) The connectors were soldered onto the driver board (e.g. PCB-connectors), the parts were then allocated on a stripboard and holes were drilled in a way that the board can be easily mounted with screws into a base made from laser-cut acrylic.
(2) The PCB-connectors for the teensy and the stepper boards were soldered on the board.
(3) All pieces were connected accordingly (Figure S6.)

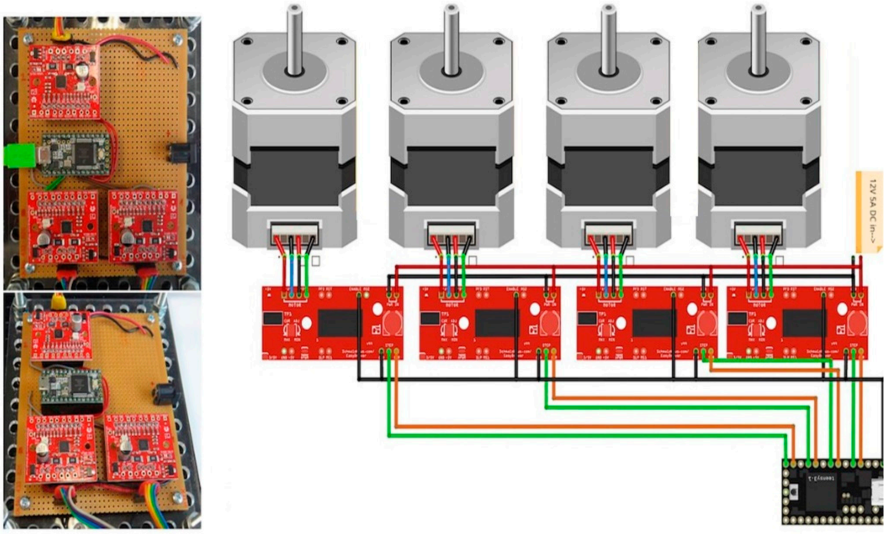

Figure S6. Motors and Electronics: Connecting board to motors: Left: Images of the electronics board with three stepper driver boards mounted. Right: Schematic diagram.

The system was designed as simple as possible, by omitting connection to MS1, MS2, MS3 (all are pulled high when not connected, i.e., the driver was set to the default 16-microstep-mode. Furthermore sleep (slp) and reset (rst) was not connected, hence these modes are automatically activated when the system is powered. The enable pin was connected to the Arduino software to be able to turn off the motors. Solely following steps had to be performed:

(1) enable (en) was was connected
(2) GND was shared between teensy and the quadstepper board(s)
(3) step-pin (stp) was activated in a way that if high for >1 microsecond, the motor will step;
(4) the direction-pin (dir) was adapted through the high/low sets which control the direction of the motors steps, which can be easily changed by reverse the connections of the leads from green, black, blue, red –> to red, blue, black, green;
(5) The microcontroller was powered by a USB connection, the motors by a 12V 5A power supply. One has to make sure that the current supplied to the motor is adjusted with the small potentiometer on the stepper driver board. On maximum setting, the chip rapidly turns hot and the motor might exhibit a strong enough torque to carry on driving beyond the end-stop, thus damaging the microscope assembly.

The motors were controlled with an Arduino compatible microcontroller and a software front end was written in Python. The Arduino IDE was used to program the microcontroller (https://www.arduino.cc/en/Main/Software). For the used "teensy" microcontrollers to be recognized by the Arduino IDE, 'Teensyduino' was installed additionally (https://www.pjrc.com/teensy/td_download.html).

Software

For communication with the microcontroller the pySerialTransfer python library was implemented. (Figure S7). The frontend was programmed in python/flask, which provides control over the stage. The python backend sends x, y and z coordinates to the Arduino firmware, which in turn controls the motor driver via the Step/Dir protocol.

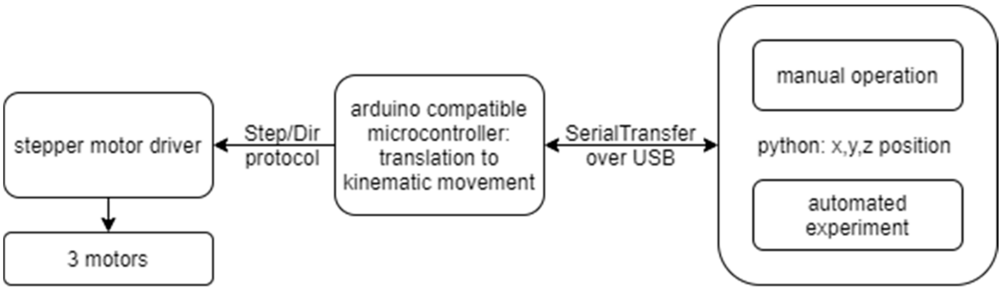

Figure S7. Schematic diagram for operating and controlling the system: The position planning and storage is by python. x-y-z positions are sent to the SerialTransfer library (python/c++) via USB and echoed back to the python program for confirmation. The microcontroller calculates the necessary movement for all three motors and sends the corresponding Step/Dir signals to the stepper motor driver, where the motor signals are generated.

For installation of the software, a copy of the github repository was created on the system (git clone https://github.com/spoc-lab/delta-microscope). All necessary python libraries can be installed with the requirements.txt file in the main directory (pip3 install requirements.txt). After the Arduino IDE has been installed, the firmware (https://github.com/spoc-lab/delta-microscope/blob/master/files/delta_microscope_arduino/delta_microscope_arduino.ino) is uploaded to the microcontroller with the Arduino software.

The python program has to be configured for use with an Arduino on the usb port "/dev/ttyACM0" and on port 80 for easy web access. The microscope software can then be started by running "python3 run.py" via console, or subsequently via integration to the boot routine of the raspberry.

After successful connection to the device hostname:port via the browser, the computer-controllable stage can be moved to any x, y and z position via login on the flask webpage (Figure S8). The webpage can either be reached under the IP address of the raspberry, or the corresponding hostname on port 80.

With + is offers a memory function, thereby storing specified positions within a workflow, which can be concatenated later to define tracks for automation of an experimental process. The camera symbol allows to take pictures. With the on/off button automated procedures can be activated or stalled. If no further experiment was specified and programmed, which can be selected under "Experiments", a "default" procedure can be started to check whether automated processing is feasible.

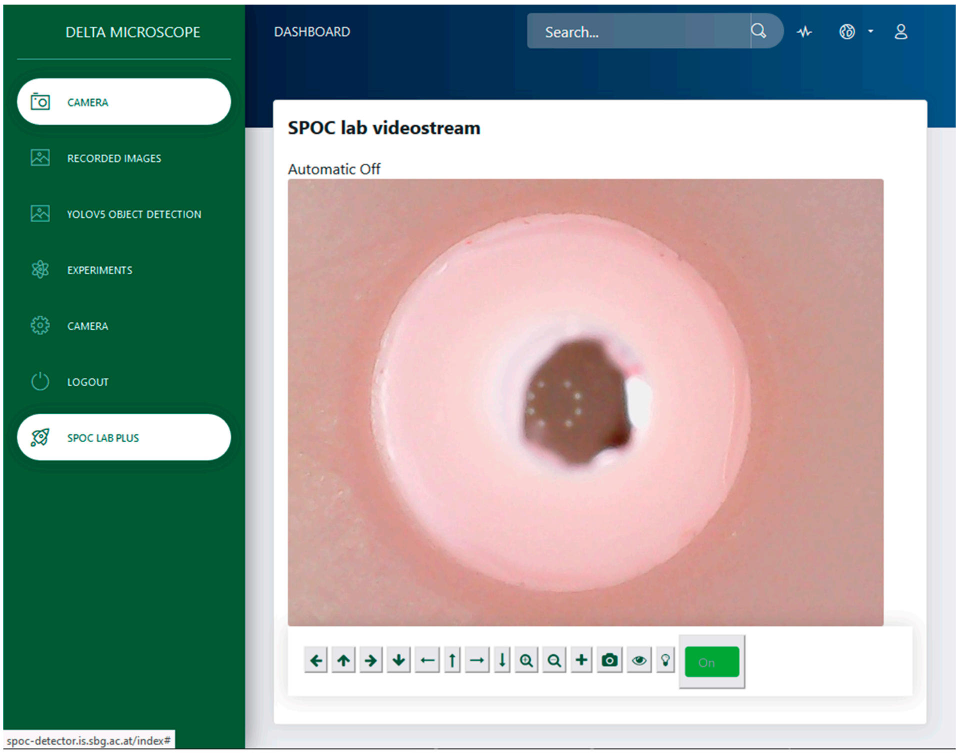

Figure S8. Web-based Interfac.: Screenshot of the created python/flask website with an integrated USB-video-microscope. The arrow buttons enable moving the stage in x, y and z direction. +/- magnifying glass symbols permit zooming. The long arrows jump the distance from one well on the chip to another. The + and – magnifier symbols are for z axis movement.

Video-microscopy

Eventually the computer-controllable stage was converted into an automated microscope. A video-microscope was incorporated at the central position underneath the stage. We successfully used a Raspberry HQ Camera v1.0 with SM1 to C-mount adapter (Thorlabs, SM1A9) to accommodate 1-inch optics connected to a Zeiss Plan 2.5 objective, as well as a low-cost USB microscope camera with integrated 40x-1000x zoom lenses. For measuring the accuracy of the stage, 0.05 mm raster was printed, placed on the microscope and an experiment with two different positions was created on the webpage and let run for 25 iterations.

The images were analyzed with a python script that found the intersections of the raster on each taken image Figure S9. Subsequently, the program compared the intersections of all images with an x,y,z position of 0 and created a boxplot of the results. This enabled us to create multiple microscope stages that could each be tested in the same manner after manufacture and assembly. The result of four of these tests gives an estimate of the mechanical accuracy of the stage and the imaging process.

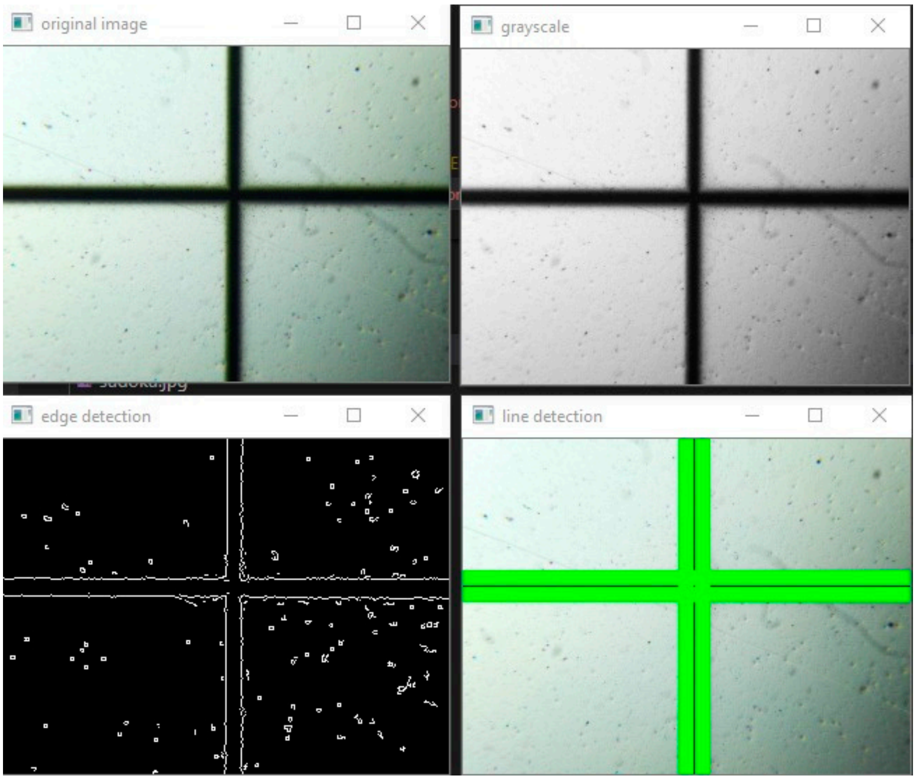

Figure S9. Image recognition. The opencv python library can be used to process the image of a printed 0.05 mm raster (https://github.com/spoc-lab/delta-microscope/blob/master/files/calibration/raster4x4.dxf). After computer vision aided automatic conversion to grayscale and edge detection, horizontal and vertical lines were detected. The crossing point of a single picture was then saved as x/y coordinate.

Supplementary 5: Immune-histological Analysis

Aggregated cells were fixed in PBS-buffer 4% formaldehyde (FA/PBS) for 30 minutes at room temperature (RT). Thereafter, specimens were washed three times with PBS and centrifuged at 2000 rpm for 5 min and prestained with Hematoxylin (Weigert) for 30 s (Roth; X906.1 and X907.1). After three rinses in PBS specimens were pelleted for 4 min at 4000 rpm. PBS was completely soaked off and 100 µL pre-warmed (~65 °C), liquid Histogel (Thermo Scientific; HG-400-012) was added, again spun down for 30 s and the chilled down at 4 °C for 20 min to allow full hardening of the Histogel. Subsequently the Histogel-spheroid-specimen was wrapped in histological Biowrap paper (Leica, 3801092) and put in a standard paraffin embedding cassette. The composite was again fixed for 1 hour in 4% para-formaldehyde/PBS, briefly dipped in PBS and then dehydrated taking it through a rising alcohol series (70% to 100% ethanol). Specimen were incubated in Roticlear for 30 min (Roth; A538.5), then in 1:1 Roticlear/Paraplast Plus (McCormick; 502004) for 30 min followed by paraffin infiltration overnight at 60 °C and a replacement of fresh liquid paraffin for another day. After paraffin infiltration the spheroid samples were embedded in molds, which thereafter were cooled to 4 °C. 3-µm thin sections were cut with the help of a microtome.

Sections were deparaffinized and rehydrated twice in Roticlear for 3 min, followed by 100% ethanol twice for 3 min, once 96%-, 80%- and 70% ethanol for 3 min and eventually in PBS buffer. Specimen were marked with a pap pen liquid blocker for easier handling and easier pipetting of solutions on the sections. Next, spheroid sections were boiled in preheated citrate buffer (10 mM, 0.05% Tween, pH=6) in a plastic cuvette at about 90 °C for antigen retrieval for 5 times, 2 min each. After each heating step, samples were cooled

down for 1 min. After the last antigen retrieval boiling step, the slides were allowed cooling down for 15 min to RT within the cuvette containing the citrate buffer. Sections were washed three times in TrisHCl-buffered saline containing 0.05% Tween (TBST) for 5 min. Sections were permeabilized by incubation in PBS containing 1% BSA, 0.4% Triton X-100 for 20 min. Thereafter, permeabilization solution was removed and sections were incubated in PBS containing 3% BSA, 0.2% Tween 20 and 0.2% Triton for 1 h at RT. The blocking solution was removed by tapping the slides. Sections were incubated with primary antibodies mouse anti human Ki67 (2 µg/mL: sc-23900 Santa Cruz Biotechnology-Inc.), or mouse anti-human-lamin A/C (2 µg/mL: sc-376248x, Santa Cruz Biotechnology-Inc.) at 4 °C overnight in a humidity chamber. Primary antibodies were diluted in blocking solution and each antibody was incubated on consecutive slides. Additional sections were treated with mouse IgG isotype controls as well as with PBS as negative controls. After antibody treatment sections were washed three times for 5 min with TBST and incubated with secondary anti mouse IgG F(ab)2 Alexa Fluor 555 antibody (4 µg/mL: 4409 Cell Signalling Technology, Inc) for 1 h at RT in a humidity chamber. Sections were washed as above and incubated with DAPI (0.2 µg/mL) for 3 min, which was rinsed off with TBST for 5 min. The slides were briefly dipped in deionized water to get rid of salt crystals, covered with EverBrite Hardset Mounting Medium (VWR; 23003) and a 0.11 µm cover slip.
